# Differences in prevalence rates of PTSD in various European countries explained by war exposure, other trauma and cultural value orientation

**DOI:** 10.1186/1756-0500-7-407

**Published:** 2014-06-28

**Authors:** Andrea Burri, Andreas Maercker

**Affiliations:** 1 Department of Psychology, University of Zurich, Zurich, Switzerland

**Keywords:** Cultural values, Mental disorder, Post-traumatic stress disorder, PTSD, War trauma, World War II, Societies, Cross-cultural assessment

## Abstract

**Background:**

Guided by previous explorations of historical and cultural influences on the occurrence of PTSD, the aim of the present study was to investigate the contributions of war victimisation (in particular, World War II) and other civil trauma on the prevalence of PTSD, as mediated by cultural value orientation. Secondary data analysis was performed for 12 European countries using data, including PTSD prevalence and number of war victims, crime victims, and natural disaster victims, from different sources. Ten single value orientations, as well as value aggregates for traditional and modern factors, were investigated.

**Results:**

Whilst differences in PTSD prevalence were strongly associated with war victim rates, associations, albeit weaker, were also found between crime victims and PTSD. When cultural value orientations, such as stimulation and conformity as representatives of modern and traditional values, were included in the multivariate predictions of PTSD prevalence, an average of approximately 80% of PTSD variance could be explained by the model, independent of the type of trauma exposure.

**Conclusion:**

The results suggest that the aftermath of war contributes to current PTSD prevalence, which may be explained by the high proportion of the older population who directly or indirectly experienced traumatic war experiences. Additional findings for other types of civil trauma point towards an interaction between value orientation and country-specific trauma rates. Particularly, being personally oriented towards stimulation appears to interact with differences in trauma prevalence. Thus, cultural value orientation might be viewed not only as an individual intrinsic process but also as a compensatory strategy after trauma exposure.

## Background

Estimates of the lifetime prevalence of post-traumatic stress disorder (PTSD) have been shown to vary considerably between countries. Data collection for a recent study on the size and burden of mental disorders in Europe, for example, revealed differences in PTSD prevalence that ranged from 0.56% to 6.67% in the general population, with one outlier country reporting a ten-fold higher prevalence [1]. Countries with the highest prevalence of PTSD were the Netherlands, the UK, France and Germany. Countries with the lowest prevalence of PTSD were Spain and Switzerland. The outlier country was Croatia.

Interpreting these differences in the prevalence of psychopathological syndromes across cultures remains a difficult task. In addition to methodological variations (e.g., assessment instruments and sample characteristics), some of the difference in PTSD prevalence estimates across nations can be ascribed to differing levels of trauma exposure. Other factors that may account for estimate variability within and across cohorts are independent of trauma exposure diversity and often remain unidentified. There is, however, an increasing appreciation for the historical, societal, and cultural influences on mental disorders, thus helping to explain PTSD prevalence, presentation and progression profiles [2,3]. PTSD has been discussed as being a culturally constructed syndrome itself, present predominately in industrialised countries of the 20th century [4,5]. In previous studies, we have proposed that value orientations in a given society or nation could serve as an independent explanatory factor for differences in PTSD presentation and prevalence [6,7].

**Figure 1:**
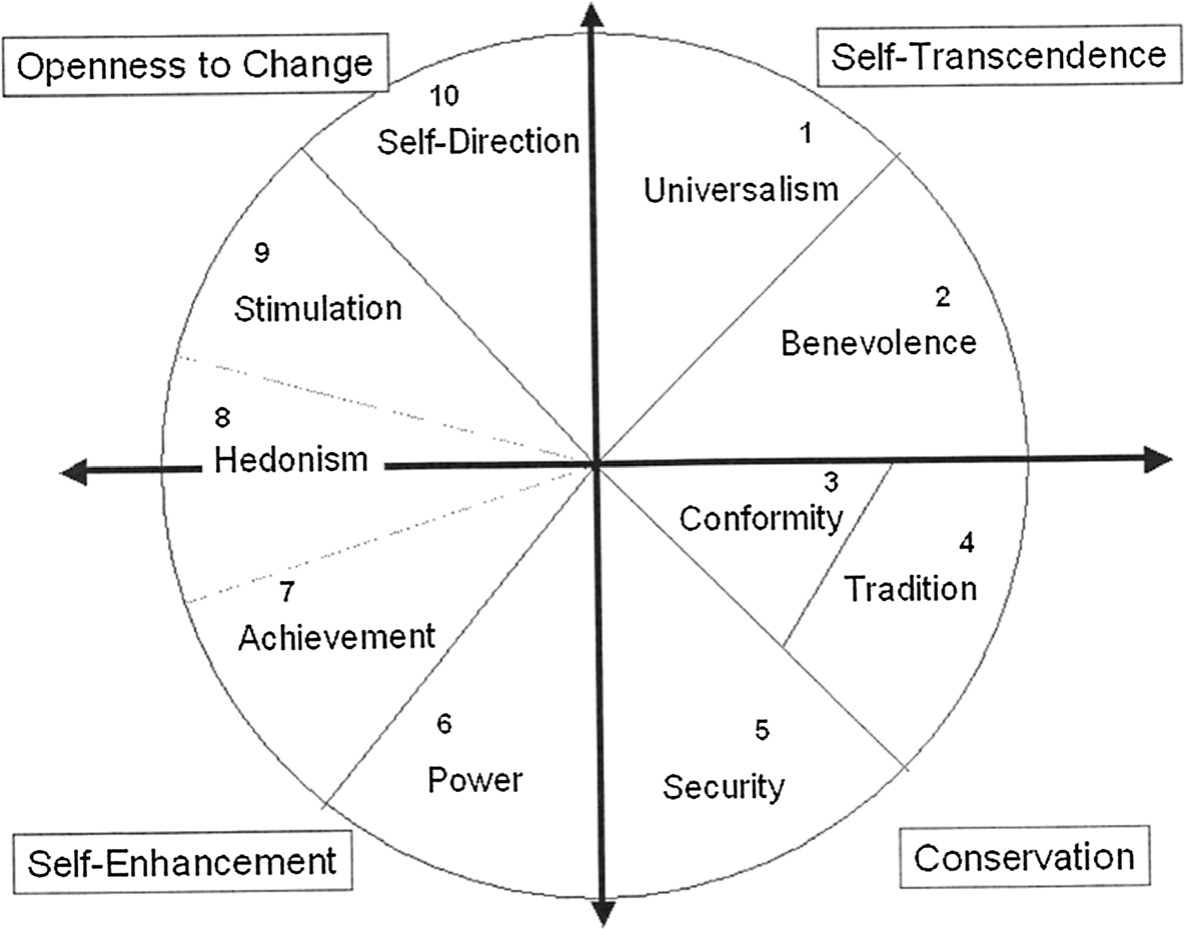
Model illustrating the relations among the ten motivational types of value as proposed by Schwartz (2006) (Illustration taken from Schwartz, Basic Human Values: Theory, Measurement, and Applications, 2006 with permission from the author).

The debate about the role and significance of value orientation in the development and expression of mental illness is not new or recent [8]. Two conceptually and theoretically different perspectives prevail: cultural relativists claim that mental illness cannot be separated from an individual’s cultural context, whereas universalists argue that biological similarity suppresses cultural influences [9]. Both perspectives, however, agree that cultural factors play a role in the development and perception of mental disorders. The main cultural factors are value orientations. According to an extensively, empirically validated theory of value orientations, ten motivationally distinct individual values can be summarised into two universally accepted dichotomous dimensions of related motivations: one dimension contrasts “openness to change” and “conservation” values, and the other dimension contrasts “self-enhancement” and “self-transcendence” values (Figure 1) [10]. As such, the theory goes beyond the simple identification of specific values and also explicates the structure of dynamic relations among the values common to culturally diverse groups. Conservation and self-transcendence can be regarded as traditional values, whereas openness to change and self-enhancement reflect modern values. Traditional values mostly represent the value orientations of less-developed societies and countries, whereas modern values correspond with and evolve alongside the achievements of modern developed countries with post-industrial economies. A description of the ten individual values can be found in Table 1.

**Table 1:** **Description of the ten motivationally distinct value orientations as described by Schwartz **[9]

**Value**	**Description**
**Self-direction**	Independent thought and action: choosing, creating exploring.
**Stimulation**	Excitement, novelty, and challenge in life.
**Hedonism**	Pleasure and sensuous gratification for oneself.
**Achievement**	Personal success through demonstrating competence according to social standards.
**Power**	Social status and prestige, control or dominance over people and resources
**Security**	Safety, harmony, and stability of society, of relationships, and of self
**Conformity**	Restraint of actions, inclinations, and impulses likely to upset or harm others and violate social expectations or norms.
**Tradition**	Respect, commitment, and acceptance of the customs and ideas that traditional culture and religion provide the self
**Benevolence**	Preserving and enhancing the welfare of those with whom one is in frequent personal contact
**Universalism**	Understanding, appreciation, tolerance, and protection for the welfare of all people and for nature

Until quite recently, scholars from the social sciences and humanities postulated that traditional value orientations were associated with less morbidity, whilst modern value orientations increased risk, especially for mental disorders [11]. However, in the case of PTSD and other stress response syndromes (e.g., adjustment disorders), several lines of evidence indicate just the opposite. With similar levels of trauma exposure, traditional value orientations were associated with greater PTSD symptom severity, whereas modern value orientations were associated with lower PTSD symptom severity [6,7]. The level of traumatic stress exposure predicted only a very small proportion of the symptom variance, particularly as shown in studies of combat-exposed war veterans [12]. Assuming that variation in the level of trauma exposure and differences in cultural value orientations determine, or at least influence, PTSD development and symptom severity, the question arises as to whether interactions between societal levels of trauma exposure and value orientations exist and to what extent such potential interactions contribute to the variability in PTSD expression and perception.

### AIM

The present study focuses on European countries. Although Europe has not been involved in any continental wars since 1945, a number of PTSD studies have suggested the presence of long-term emotional and psychological impact (i.e., up to 40 years after the war experiences) of the Second World War (WWII) on the population, even on second and third generations [13-15].

The first aim of this study was to explain the variability in PTSD prevalence reported across a number of European nations by the effects of trauma and value orientations. As primary predictors, four main types of trauma exposure were examined: war fatalities, crime victimisation, natural disasters, and road fatalities. Because only a fraction of the variability in PTSD prevalence can be attributed to different levels of trauma exposure, the second aim of the present study was to explore value orientations as determinants of PTSD prevalence disparity, beyond the level of trauma exposure. More specifically, based on findings from our own previous research, we expected “modern values” to be associated with less severe PTSD and “traditional values” to be associated with more severe PTSD, which is in contrast to a layperson´s expectations [6]. In this context, an antecedent note of caution is appropriate: the primary determinants included in this study are estimates originating from various sources, which have been jointly investigated in these secondary analyses. Although all sources are valid and representative and all estimates (or proxies of trauma exposure) have been assessed using solid methodologies, the unknown error variance of each parameter needs to be considered when interpreting the results. Therefore, the explorative character of this study has to be considered.

## Methods

### Samples

For this study, we used data from the European Size and Burden of Disorders of the Brain study, organised by the European Brain Council (EBC) (see Table 2) and supplemented by World Mental Health Survey data [1,16,17]. Data analysis was conducted only for countries that had data available for population-based representative PTSD estimates, information on value orientation, and estimates of accidental versus interpersonal trauma exposure. These criteria resulted in a total of 11 European countries. Each data source relied on different sample sizes.

**Table 2:** Descriptives for PTSD and traumatic events for 11 countries included in the study

**Country**	**PTSD Prevalence (%)**	**Rates of traumatic events**			
		War victims*	Crime victims**	**Natural disasters**^**†**^	Road fatalities**
Belgium	0.76	.007	9.6	4.4	10.1
France	2.32	.013	5.7	7.8	6.9
Germany	2.31	.009	7.5	5.5	4.5
Italy	0.73	.009	4.5	7.8	8.7
Netherlands	3.30	.014	2.5	3.9	4.1
Croatia	6.67	.100	14.2	-	10.4
Spain	0.56	.000	5.1	2.9	6.9
Switzerland	0.70	.000	4.2	2.4	4.7
UK	3.00	.009	7.7	3.7	3.6
Bulgaria	0.94	.002	1.7	1.3	13.5
Romania	0.38	.012	1.4	5.1	12.7

*In proportion to total inhabitants.

**In proportion to million inhabitants.

^†^Prevalence of lifetime exposure to natural disaster (Kessler et al., 2011).

### Measures

#### ***PTSD Prevalence***

In 2003, the European Research Council (ERC) and the European College of Neuropsychopharmacology (ECNP) launched a Europe-wide interdisciplinary project aimed at assessing the economic burden of neurological and mental disorders [1,18,19]. In 2009, an updated and improved program was launched. This program provided consolidated estimates for the 12-month prevalence of mental disorders, including PTSD, for all EU countries. For our analysis of the relationship between PTSD and rough estimates of trauma, prevalences of PTSD from the following studied countries were included: Belgium, France, Italy, Spain, Germany, Switzerland, the UK, the Netherlands, Bulgaria, Romania, and Croatia [18-24]. Note that in the original EBC publication, the Croatian prevalence of PTSD was reported as a proxy for the neighbouring EU-country Slovenia [18,19].

#### ***Value orientation***

Data on value orientation were taken from the European Social Survey (ESS), a biennial cross-sectional survey initiated in 2001, which was designed to chart attitudes, beliefs and behaviour patterns of Europe’s diverse populations. The survey includes 21 questions based on Schwartz’s original 40-item portrait values questionnaire (PVQ), which was developed to assess value orientations [20]. Schwartz’s value typology is grouped into ten value types: security, conformity, tradition, benevolence, universalism, power, self-direction, stimulation, hedonism, and achievement. The value structure is defined by two different sets of values: individual interest (power, achievement, hedonism, stimulation, and self-direction) and collective interest (benevolence, tradition, conformity, universalism, and tradition). As mentioned before, the ten motivationally distinct values can be summarised into four dimensions: “openness to change”, “conservation”, “self-enhancement”, and “self-transcendence” values. The four dimensions, also called “higher-order values”, express a broad motivational goal that is shared by the basic values that compose it. In the “self-enhancement vs. self-transcendence” dimension, values of power and achievement oppose values of universalism and benevolence. Both former values emphasise the pursuit of self-interests, whereas both latter values involve concern for the welfare and interests of others. In the “openness to change vs. conservation” dimension, values of self-direction and stimulation oppose values of security, conformity and tradition. Both former values emphasise independent action, thought and feeling, as well as a readiness for new experience, whereas the three latter values emphasise self-restriction, order and resistance to change. Hedonism shares elements of both openness to change and self-enhancement but, in most cases, hedonism is closer to openness to change. The individual values can further be assigned to two main factors: tradition (security, conformity, tradition, power, and achievement) and modern (hedonism, benevolence, universalism, self-direction, and stimulation).

Each value type was assessed with two questions, except for universalism, which was assessed with three questions due to its broad content. Respondents were asked to rate the extent to which the description of a fictitious person corresponded to their own attitudes and behaviours. Response options were on a six-point Likert-type scale, ranging from “very much like me” (1) to “not like me at all” (6). Evidence for the validity of the theoretical and content-related structure of Schwartz’s typology has been demonstrated previously in samples from 67 nations and recently in data from 38 countries [10,21,22].

Data collected in the ESS can be freely accessed from their webpage, http://www.europeansocialsurvey.org, which includes a brief description of score computation for the Human Value Scale. For more information on the assessment of value orientation within the context of the ESS, see Davidov 2010 [23].

#### ***Level of trauma exposure***

Potentially traumatic events can be divided into two basic groups: intentional or interpersonal and accidental. The intentional or interpersonal group includes war, abuse, and violence. The accidental group includes earthquakes, hurricanes, floods, fires and technical catastrophes. To assess the degree of exposure to traumatic events, estimates of various types of intentional events and natural disasters were included in this study. The number of war deaths during WWII for each of the eleven countries included in this study was taken from Preger and Mourik [24]. For each country, we then calculated a proportion measure representing the number of war victims per million inhabitants, based on population estimates for 1945. For Croatia, the war deaths were calculated using the Yugoslavian WWII data (proportion of Croatians out of the Yugoslavian total population) plus the war deaths from the Balkan wars in the early 1990s, resulting in 1.5 million deaths during WWII and 12’130 during Yugoslavian wars [25].

Information on the number of crime victims was taken from Eurostat, a directorate-general of the European Commission, which provides statistical information on the Institutions of the European Union for a wide range of variables. The figures used in this study represent the estimates of crime victims per million inhabitants for the year 2008 [26]. The category “crime victims” includes homicide, violent crime, robbery, and domestic burglary.

For accidental trauma, exposure rates for natural disasters were taken from the WHO World Mental Health Survey (WMH survey; n = 56’872), which is a series of epidemiologic surveys carried out in a number of countries throughout the world to allow comparison of prevalences and correlates of mental disorders [27]. Of the original 28 countries that completed the WMH surveys, we selected the eleven countries that had data available for PTSD prevalence. In these surveys, exposure to natural disasters was assessed using the PTSD section of the WHO Composite International Diagnostic Interview Version 3 (CIDI) [28]. For more information on the WMH survey and data assessment, see Kessler and Üstün [17]. The number of road fatalities was based on the WHO Global Status Report on Road Safety from 2009, which represents the estimates of road fatalities per million inhabitants for the year 2008 [29].

### Statistical analysis

Statistical methods appropriate for small data sets were chosen. First, simple correlations were calculated between PTSD prevalence and estimates of accidental and intentional/interpersonal disasters and value orientation. For correlation analyses, Spearman’s rank correlation coefficients (rS) were used. Spearman correlations were also calculated to investigate the relationship between PTSD and the four trauma exposure levels and value orientations. The advantages of using Spearman's rank correlations, rather than the more common product moment correlations, are that Spearman’s rank correlation is unaffected by the distribution of the population and can be used with very small sample sizes [30]. All analyses were carried out separately on the ten individual values, the four dimensions, and the two main factors. Because the small sample size restricted significance testing, correlations were considered statistically significant when p < 0.05 and were referred to as “substantial” when rS > 0 · 3, even if p > 0.05.

Multiple linear regression analyses were carried out to investigate the moderating effect of value orientations, as independent variables, on the relationship between PTSD prevalence and the four categories of trauma exposure level. In model 1, the main effect of the two main aggregate factors, traditional values and modern values, was investigated. Model 2 included the main effect, as well as the interaction effect of the two aggregate factors. In model 3, two specific individual values, conformity and stimulation, and their interaction with trauma exposure were included. These two values were chosen because of their high correlation with PTSD prevalence and their importance in the two-dimensional concept of value orientation. Stimulation and conformity best represent the dimensions of “openness to change” and “conservation”, respectively, without overlap of the other dimensions, therefore insuring more “homogeneity” in the concept.

Multiple linear regression modelling assumes a normal distribution for the error terms, which was granted in our dataset. Beta values represent standardised betas, indicating how many standard deviations (SDs) the dependent variable (PTSD prevalence) will change per SD increase in the predictor variable(s). Given our small sample size of only twelve observations (i.e., countries), conventional significance levels may largely be ignored when interpreting the data and, instead, attention given to the relative magnitude of the obtained betas – where indicated [31]. This approach is recommended for research relying on small samples. The missing prevalence data for natural disasters for Croatia was imputed using multiple imputation, which is a simulation-based approach that replaces missing values with multiple sets of simulated values. This method further adjusts the obtained parameter estimate for missing-data uncertainty [32]. Because Croatia was a statistical outlier in terms of PTSD prevalence and was also more likely to report aftermath effects of the Balkan war, rather than WWII, analyses were conducted both with and without Croatia. All data handling and descriptive analyses were completed using STATA (StataCorp. 2009. Stata Statistical Software: Release 11. College Station, TX: StataCorp LP.).

## Results

### PTSD prevalence and trauma exposure rates

PTSD prevalence and trauma exposure rates for the twelve nations are reported in Table 2. Strong statistically significant associations were observed between PTSD prevalence and war victim rate (rS = 0.87, p < 0.001) and between PTSD prevalence and crime victim rate (rS = 0.69, p < 0.05; Table 2). No statistically significant associations were found between PTSD prevalence and accidental trauma events, including natural disasters and road fatalities.

### Associations between PTSD prevalence, value orientation and trauma exposure

In the first step, correlations between PTSD and the individual values were calculated, and in the second step, correlations between PTSD and the two aggregate factors described in the methods section (i.e., the two factors related to tradition and modern value orientation) were calculated (Table 3). For the association of the individual values with PTSD, the highest correlation was found between the modern value “stimulation” and PTSD prevalence (rS = 0.44) and the lowest correlation was found between the modern value “benevolence” and PTSD prevalence (rS = 0.002). For the aggregate factors, the factor reflecting a modern value orientation correlated substantially stronger with PTSD prevalence compared with the factor reflecting a traditional value orientation (rS = 0.28 vs. rS = 0.13). Overall, the proxies of trauma exposure correlated highest with the individual values that represent a modern value orientation, with significant correlations found between war victims and “stimulation” (rS = 0.73, p < 0.05) and road fatalities and “self-direction” (rS = -0.80, p < 0.01).

**Table 3:** Correlations between PTSD prevalence and prevalence estimates of intentional and accidental traumatic events and value orientations

	**PTSD Prevalence**	**War victims**	**Crime victims**	**Natural disaster**	**Road fatalities**
**PTSD Prevalence**	**-**	0.87***	0.69*	0.29	-0.16
**War victims**		**-**	0.73*	0.57*	0.22
**Crime victims**			-	0.48	-0.08
**Natural disaster**				**-**	0.04
**Road fatalities**					**-**
**Individual values**					
**Security**	0.35	0.57	0.46	0.19	0.50
**Conformity**	-0.32	-0.29	-0.25	-0.33	-0.37
**Tradition**	-0.09	-0.17	-0.24	0.16	-0.16
**Benevolence**	0.002	-0.08	0.20	-0.54	-0.33
**Universalism**	-0.36	-0.48	-0.42	0.07	-0.27
**Self-direction**	-0.14	-0.39	-0.47	-0.45	-0.80**
**Stimulation**	0.44	0.73*	0.62^†^	0.15	0.59^†^
**Hedonism**	-0.04	0.19	0.04	0.48	0.44
**Achievement**	-0.31	-0.37	-0.35	-0.01	-0.17
**Power**	-0.25	-0.48	-0.37	0.00	-0.40
**Aggregated values**					
**Traditional**	0.13	0.36	0.25	-0.00	0.24
**Modern**	0.28	0.56	0.36	0.23	0.44

^†^ = p < .1, *p < 0.05; **p < 0.01; ***p < 0.001.

The strongest positive correlations were between the individual value “stimulation” and the exposure types “war victims” and “crime victims” (rS = 0.73, p < 0.01 and rS = 0.62, p < 0.1, respectively; Table 3). The strongest negative correlations were between the individual value “self-direction” and road fatalities (r = 0.80, p < 0.005) and between “universalism” and “power” and all trauma exposure types, except natural disasters (rS’s ranging from 0.25 to 0.48). Overall, consistent patterns of negative correlations were observed for “conformity”, “universalism”, “self-direction”, “achievement” and “power” with all trauma exposure types, whereas consistent positive correlations were only observed for “stimulation”, “hedonism” and “security”.

### Predicting prevalence differences by aggregate and/or individual value orientations

Multiple linear regression of PTSD prevalence indicated that a model including war victims and both aggregate factors of modern and traditional value orientation best explained the variance in PTSD prevalence, compared with regression models based on the three other trauma exposure proxies (R^2^ of 0.79 vs. R^2^’s of -0.39, 0.15, and -0.39, respectively; Table 4). When including “specific exposure type x aggregate factor interactions” in the models, variance in PTSD prevalence explained by the war victim model was even higher, reaching R^2^ of 0.82. Similarly, a significant increase in variance explanation was observed for the models based on crime victims and road fatalities, whereas no such drastic increase was observed for the natural disaster model. To further interpret these relationships and interactions, post-hoc visual examinations were conducted. These visual examinations revealed that lower crime rates and lower modern values predicted lower PTSD prevalence, whereas lower crime rates and higher modern value orientation predicted higher PTSD prevalence (beta =3.17, p < 0.05).

**Table 4:** Regression analyses of PTSD prevalence, proxys of trauma exposure and value orientation using different models

	**War victims**			**Crime victims**			**Natural disaster**			**Road fatalities**		
	**Beta**	**p**	**R**^**2**^	**Beta**	**p**	**R**^**2**^	**Beta**	**p**	**R**^**2**^	**Beta**	**p**	**R**^**2**^
**Model 1**			0.79*			**-0.39**			**0.15**			**-0.39**
SVE	1.01	0.003		-.077	0.86		.20	0.67		-.04	0.93	
Aggregated Factor “Traditional”	.03	0.92		-.34	0.67		-.11	0.88		-.38	0.64	
Aggregated Factor “Modern”	-.35	0.35		.57	0.49		-.49	0.53		.63	0.49	
**Model 2**			**0.82**^†^			**-0.30**			**-1.06**			**0.36**
SVE	5.79	0.14		3.35	0.04		-1.26	0.73		-2.5	0.08	
Aggregated Factor “Traditional”	.63	0.43		-2.12	0.09		3.31	0.76		.40	0.82	
Aggregated Factor “Modern”	-.33	0.59		3.17	0.04		-4.21	0.69		-2.87	0.16	
SVE x Aggregated Factor “Traditional”	-3.99	0.59		.98	0.41		-5.59	0.76		-2.02	0.58	
SVE x Aggregated Factor “Modern”	.98	0.89		-4.4	0.044		7.62	0.73		6.5	0.17	
**Model 3**			**0.79**^†^			**0.35**			**0.32**			**0.76**^†^
SVE	2.82	0.48		.65	0.41		-2.54	0.28		-2.53	0.02	
Conformity	.04	0.87		-.64	0.14		-1.96	0.21		-4.87	0.12	
Stimulation	-.20	0.73		.81	0.09		-2.16	0.21		-1.62	0.09	
SVE x conformity	.08	0.73		1.28	0.11		1.88	0.21		4.68	0.12	
SVE x stimulation	-1.76	0.68		-1.96	0.07		2.80	0.31		4.01	0.02	

Note: SVE = specific variable expression of the main variable included in the model (i.e. war victims, crime victims, natural disaster, road fatalities, respectively); R^2^ = variance expressed by the model; beta = standardized betas.

^†^p-valuesfor whole model testing= p< .1, *p < 0.05, **p <0.001.

**Figure 2:**
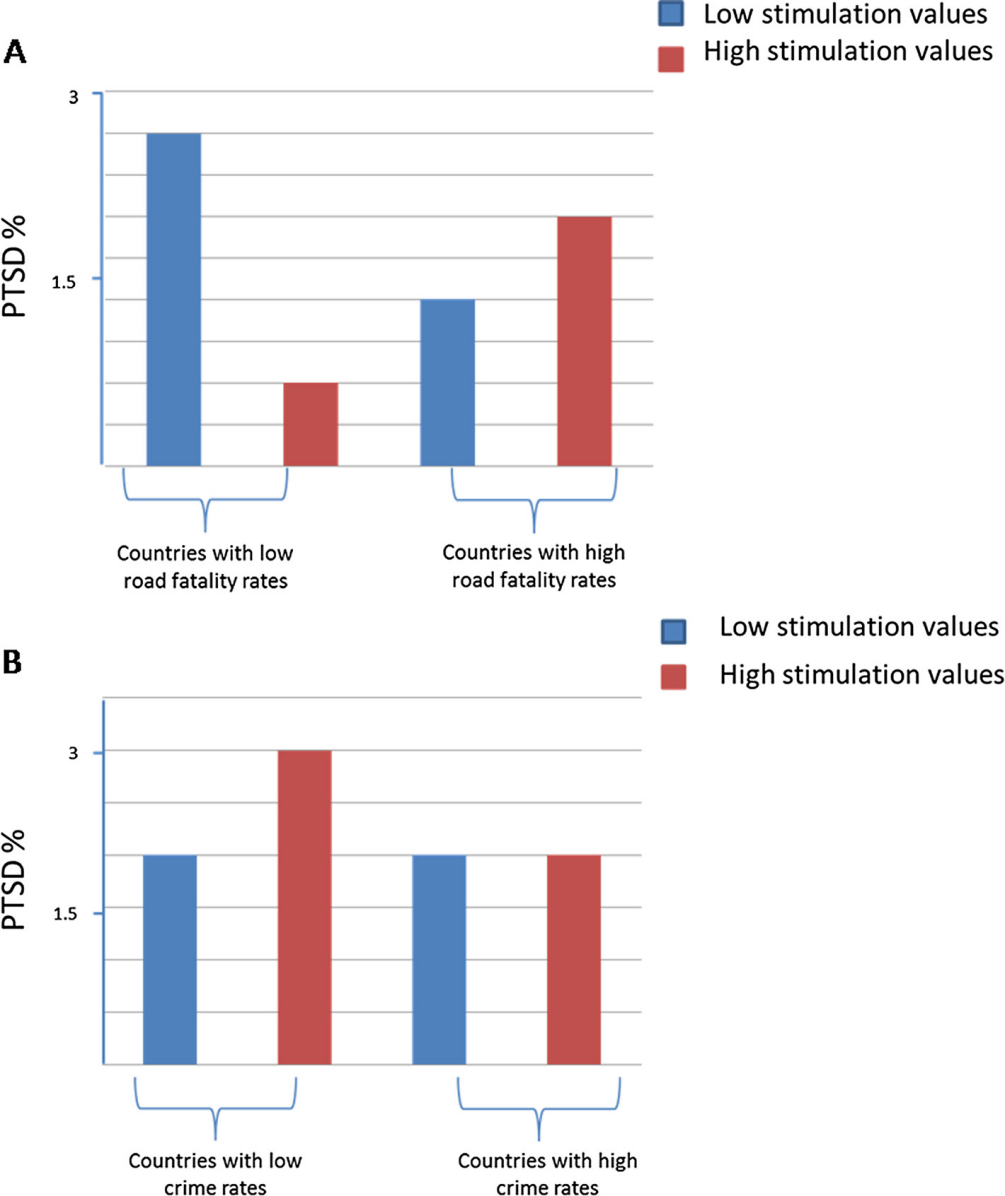
**AB Interaction effects in predicting PTSD. A)** Interaction effects between crime rates and the individual value of ‘stimulation’. **B)** Interaction effects between road fatality rates and the individual value of ‘stimulation’.

Because the possibility of co-dependency of values within the aggregate factors could not be excluded, a second step that replaced the aggregate factors with two individual values most representative of modern and traditional value orientations, i.e., stimulation and conformity, was included in the model. A model including “specific exposure type”, “conformity” (as representative of traditional value orientation), “stimulation” (as representative of modern value orientation), and the interaction terms showed the highest variance explanation across all four types of trauma exposure proxies (Table 4). However, in contrast to “stimulation”, “conformity” did not significantly contribute, either as a main or as an interaction effect, to the prediction of PTSD. When analysing the data including crime victims, a picture similar to the aggregated modern values emerged (Figure 2). Further inspection of the regression including road fatalities revealed that higher road fatalities and lower stimulation orientation predicted lower PTSD prevalence.

As previously mentioned, all analyses were additionally conducted excluding Croatia. However, no significant deviations from the initial results were detected. Therefore, only results from the full dataset are presented and discussed.

## Discussion

Overall, our findings revealed a significant predictive effect of value orientation on PTSD prevalence in a sample of 11 European countries. In particular, low crime rates and high modern value orientation (i.e., aggregate factor) predicted higher PTSD prevalence. This result was supported by the model that substituted “stimulation” for the aggregate factor as representative of modern value orientation, which excluded the possibility of co-dependency of values within the aggregate factor. In this model, the main effect and interaction effect of stimulation accounted for the highest variance in PTSD prevalence, compared with the individual value “conformity”, as representative of traditional values.

Whilst much research effort has been invested in identifying and analysing the relationship of numerous biological and psychological factors and their interplay with PTSD, the variability of PTSD prevalence in relation to diverse cultural and societal influences has so far been under researched. To fill this gap, the aim of the current study was to explore these potential societal influences, including historical and cultural factors, using value orientations. Supported by findings from previous research by our group, we hypothesised that disparities in PTSD prevalence across European countries are related to differences in value orientations and in trauma exposure rates [6,7].

Our main finding showed a strong association between war fatalities and PTSD prevalence rates across the 11 European countries included in this study. This association remained stable in subsequent multivariate analyses, even after including value orientations. For most countries, the war death rates taken as a proxy for trauma exposure were based on WWII victims. A number of previous studies have shown that affected people still suffer from PTSD or present with severe PTSD symptoms 60 years post WWII (the original studies on PTSD in the different countries were conducted between 1998 and 2005). Living through WWII and its aftermath involved being confronted with a number of distressing life situations, such as direct personal exposure to combat or bombings, traumatic experiences during migration or displacement, or more ‘indirect’ effects resulting from consequential childhood hardships (such as starvation, cold, migration, or displacement), which can be triggered by subsequent traumatic events into full-blown PTSD [33]. The latter effect is known and discussed as a secondary or tertiary traumatisation effect [34].

With regard to aggregate or individual value orientations, correlational findings between number of war victims and value orientation essentially confirm previous study findings that reported modern values, especially stimulation, were positively associated with PTSD and even more so with trauma exposure rates [6,7]. Stimulation, as a modern value orientation, is distinguished by seeking excitement, novelty, and challenge in life, by daring and by living a varied and exciting life. In our study, the expression of this value was stronger in nations who reported a higher number of traumatic events and increased PTSD rates. To the best of our knowledge, no social-psychological or sociological explanation exists for this association between population-level value orientation and PTSD prevalence. However, on an individual level, the relationship between trauma exposure and/or PTSD and the personality trait of “sensation seeking” has been debated and substantiated for its pathological effect [35,36]. Sensation seeking describes an urge to pursue novel, intense and complex sensations and experiences, and the willingness to take risks for the sake of such experience. As such, it is comparable to the population-level value of “stimulation”. Research evidence has suggested an association between sensation seeking and PTSD, although not in the direction previously predicted from the compulsive exposure hypothesis (i.e., high sensation seeking is associated with PTSD). In a study conducted by Joseph et al., the investigators explored the association between PTSD and impulsivity (parameterised as consisting of the two components impulsiveness and venturesomeness). The authors found no relationship between PTSD and venturesomeness, but a significant association between PTSD and impulsiveness [36].

Further interesting results were observed related to the other exposure types, crime, natural disasters, and road fatalities, investigated in this study. Crime and road fatality related trauma exposure and PTSD was predicted not only by the exposure rates themselves but also by the cascades of main and interaction effects of value orientation x trauma exposure. For the trauma proxy of crime victims, a modern value orientation in society predicted the development of PTSD. When taking the interaction effect between modern values and trauma exposure into account, an additional increase in PTSD rates was observed. In terms of the modern value “stimulation”, this means that higher stimulation values predicted higher PTSD. This association was strengthened when including the interaction effect between stimulation and trauma exposure (i.e., crime rates). The latter may be particularly true in countries such as Bulgaria or Rumania, which have low rates of officially reported crimes (which formed the basis for our data analyses), but a much higher true figure of crimes [37]. A tentatively significant, but reverse, finding was found for road fatalities, in which stimulation as a modern value predicted lower PTSD rates, and in which lower crime exposure combined with lower stimulation values predicted higher PTSD. The UK, the Netherlands and Germany showed comparably low rates of road fatalities, and citizens with low modern stimulation orientation were consistently more prone to develop PTSD when exposed to road accidents, compared with citizens with traditional value orientations (e.g., conformity).

To interpret the differential findings described in the preceding two paragraphs, a simple distinction may help: war and crime-related victimisation belong to the category of interpersonal traumatic exposure. These traumas are considered to be especially anomalous and aberrant by the victims, who subsequently look for culprits and perceive the world as meaningless and incomprehensible, and consequently change their world-views [38]. In contrast, accidental trauma, such as natural disasters or road fatalities, is perceived as inevitable and predestined, and provokes fear and caution [39]. Because of this, interpersonal trauma is more likely to negatively interact with modern values, such as stimulation, compared with accidental trauma that will correlate positively. In other words, individuals living in countries where modern value orientations predominate are more likely to suffer traumatic stress due to interpersonal trauma exposure, whereas in countries with traditional value orientations, accidental or coincidental trauma might lead to substantial stress.

When interpreting the influence of cultural values on trauma and trauma consequences, additional aspects such as whether and how trauma exposure itself might shape cultural orientation need to be considered. Research on changes in pro-social attitudes after traumatic events, e.g., natural disasters [40] and terroristic attacks [41], does suggest such effects. Furthermore, our results suggest that the effects of traumatic events can last more than 40 years, further highlighting the potential intergenerational effect of trauma sequelae, and how such stress-related consequences can be present in offspring of war combat participants [42].

Importantly, the results of this study need to be interpreted in view of certain study limitations. First, because information on value orientation and corresponding PTSD prevalence was not available for all European countries, resulting in 12 countries included in this study, restricted power in statistical testing needs to be addressed. To address this issue, statistical methods were chosen that are proven to be highly satisfactory when sample sizes are small. Nevertheless, replication of the study in other populations with information available on war victimisation (in particular, World War II) and other civil traumas, as well as on PTSD prevalence and value orientations, should be considered.

Second, the high PTSD prevalence in some of the countries included in this study might not result from war deaths alone but also from recent war survivors, e.g., British and French soldiers that were involved in the Gulf War, where most likely other soldiers from other countries were not involved. Although this heterogeneity in sample characteristics of war victimisation might have contributed to variation in PTSD figures, we highlight the fact that several other sources of traumatisation (road fatalities, natural catastrophes and crime victimisation) were included in the study; thus, allowing for a comprehensive understanding of population traumatisation and value orientations, and their associations with PTSD.

Third, the fact that the primary determinants included in this study are estimates originating from various sources that have been jointly investigated in these secondary analyses should be considered. Although all sources are valid and representative, and estimates (or so called proxies of trauma exposure) have been assessed using solid methodologies, the unknown error variance of each parameter needs to be considered when interpreting the results. Furthermore, aggregate data have a high risk of misinterpretation as often described by “Simpsons Paradoxon”. It describes a case of probability statistics in which a trend that appears in different groups of data disappears when these groups are combined, and the reverse trend appears for the aggregate data. This result is often encountered in social science and medical science statistics and is particularly confounding when frequency data are unduly given causal interpretations. Therefore, replication of the results in other population using study-specific, non-aggregated data is needed.

## Conclusion

In conclusion, despite shortcomings of sample size and secondary inhomogeneous data sources, the present study tentatively suggests that the currently observed cross-national differences in PTSD prevalence can be explained by long-term WWII consequences. The findings further propound a relationship between value orientations, specifically modern values such as stimulation, and cross-national rates of traumatisation and PTSD symptomatology. The pursuit of novel, intense and exciting sensations might therefore not only be regarded as an individual intrinsic process or trait but also as a coping strategy for the terror and emotional sequelae of repetitive trauma exposure. Whether these processes represent a sustainable, additional explanatory model for the variability in PTSD prevalence needs to be further explored.

## Competing interest

The authors report no conflicts of interest.

## Authors’ contribution

AB carried out the analyses and drafted the manuscript. AM participated in the design of the study, helped interpret the results and drafted the manuscript. Both authors read and approved the final manuscript. The corresponding author further confirms that he had full access to all data in the study and that he had the final responsibility for the decision to submit the manuscript for publication.
